# Downregulation of Profilin-1 Expression Attenuates Cardiomyocytes Hypertrophy and Apoptosis Induced by Advanced Glycation End Products in H9c2 Cells

**DOI:** 10.1155/2017/9716087

**Published:** 2017-11-07

**Authors:** Dafeng Yang, Ya Wang, Minna Jiang, Xu Deng, Zhifang Pei, Fei Li, Ke Xia, Lingyan Zhu, Tianlun Yang, Meifang Chen

**Affiliations:** ^1^Department of Cardiology, Xiangya Hospital, Central South University, Changsha, Hunan 410008, China; ^2^Department of Cardiology, Third Xiangya Hospital, Central South University, Changsha, Hunan 410006, China; ^3^Department of Endocrinology, The First Affiliated Hospital of Nanchang University, Jiangxi 330006, China; ^4^Department of Geriatric Medicine, Xiangya Hospital, Central South University, Changsha, Hunan 410008, China

## Abstract

Cardiomyocytes hypertrophy and apoptosis induced by advanced glycation end products (AGEs) is the crucial pathological foundation contributing to the onset and development of diabetic cardiomyopathy (DCM). However, the mechanism remains poorly understood. Here, we report that profilin-1 (PFN-1), a well-known actin-binding protein, serves as a potent regulator in AGEs-induced cardiomyocytes hypertrophy and apoptosis. PFN-1 was upregulated in AGEs-treated H9c2 cells, which was associated with increased cardiomyocytes hypertrophy and apoptosis. Silencing PFN-1 expression remarkably attenuated AGEs-induced H9c2 cell hypertrophy and apoptosis. Mechanistically, AGEs increased PFN-1 expression through elevating ROS production and RhoA and ROCK2 expression. Consequently, elevated PFN-1 promoted actin cytoskeleton disorganization. When either ROS production/ROCK activation was blocked or cells were treated with Cytochalasin D (actin depolymerizer), H9c2 cells were protected against AGEs-induced cardiac myocyte abnormalities, concomitantly with downregulated expression of PFN-1 and improved actin cytoskeleton alteration. Collectively, these data suggest that PFN-1 may play an important role in AGEs-induced hypertrophy and apoptosis in H9c2 cells.

## 1. Introduction

Cardiomyocytes hypertrophy and apoptosis is the crucial pathological foundation contributing to the onset and development of diabetic cardiomyopathy (DCM), which accounts for the major cause of disability and death among diabetic patients [1, 2]. Unfortunately, there is no efficient therapy to protect against DCM. Advanced glycation end products (AGEs), a group of heterogeneous compounds robustly formed under the condition of long high glucose, have been proven to be a pivotal driving force of the development and progression of DCM [3, 4]. Previous studies have demonstrated that the speed of AGEs accumulation in diabetic tissues, such as cardiomyocytes in heart failure, is much faster than in normal tissues [3, 5–7]. In addition, AGEs from the diet have also been proven to be an important factor promoting the progress of inflammation and oxidative stress [8, 9]. Recently, it was reported that binding of AGEs to their membrane receptor (receptor for AGEs, RAGE) increases the production of reactive oxygen species (ROS) and induces further production of AGEs, which subsequently promotes cardiomyocytes hypertrophy, apoptosis, and myocardial fibrosis, ultimately leading to heart failure [10–13]. More importantly, this process is prolonged and irreversible and this phenomenon is called “metabolic memory.” Therefore, the levels of AGEs are thought to be a new biomarker using as a diagnostic and prognostic tool to evaluate organ damage in diabetic patients [14, 15] and become an attractive pharmacologic target for diabetic patients [16]. However, the mechanism of how AGEs cause cardiomyocytes apoptosis, hypertrophy, or fibrosis contributing to DCM remains incompletely understood.

The cytoskeletal structure of myofibril is highly differentiated and forms the scaffold sustaining cardiomyocytes to perform contractile function [17–19]. Moreover, the dynamic cytoskeleton has been shown to play a vital role in mediating signaling pathways and regulating gene expression [20, 21]. Thus, cytoskeletal alterations are thought to be both a cause and a consequence of contractile dysfunction and myocardial remodeling resulting in heart failure. It is well known that small GTPase RhoA/Rho-associated coiled-coil-containing protein kinase pathway (RhoA/ROCK) is a crucial network involved in regulating actin cytoskeleton organization and regulates transcription factors leading to cellular hypertrophy [22–24]. However, the link between RhoA/ROCK pathway and actin cytoskeletal alteration remains largely obscure in DCM. Profilin-1 (PFN-1) is a well-known conserved actin-binding protein that plays an essential role in the regulation of cytoskeleton by promoting actin polymerization and remodeling [25]. Although this is consistent with PFN-1 being an essential gene, the mechanistic basis of the opposing functions of PFN-1 in DCM is still not fully elucidated to date. Previous studies found that elevated PFN-1 expression contributed to pathological cardiac hypertrophy and fibrosis by increasing actin polymerization in spontaneously hypertensive rats (SHRs) [26]. Recently, it was reported that PFN-1 localized to the Z-line of myofibrils under normal condition and accumulated near the M-line when overexpressed, which induced cardiomyocytes hypertrophy and dysfunction through elongated sarcomeres, myofibrillar disorganization, and sarcomeric disarray [27]. In our previous study, we found that PFN-1 plays an important role in AGEs-induced endothelial abnormalities via promoting cytoskeleton rearrangement and redistribution [28] and silencing the expression of PFN-1 attenuated AGEs-induced myocardium injury including cardiac hypertrophy and fibrosis* in vivo* [29]. However, the effect of PFN-1 on cardiomyocytes hypertrophy and apoptosis* in vitro*, especially under the stimulation of the important pathogenic factor AGEs, is still not clear. Thus, this study aims to clarify the possible role of PFN-1 in myocardial injury induced by AGEs in neonatal rat heart-derived H9c2 cells.

## 2. Materials and Methods

### 2.1. Cell Culture and Adenoviral Infection

H9c2 cells (CRL-1446, ATCC) were cultured in DMEM (HyClone, USA) containing 10% FBS (Evergreen Co. Ltd., China), 100 U/ml penicillin, and 100 *μ*g/ml streptomycin and grown in a humidified atmosphere of 5% CO_2_ in air at 37°C. For cardiac differentiation, H9c2 cells were cultured in 6-well plates at a density of 5 × 10^4^ cells/ml supplemented with 10% FBS; after 70–80% confluence, the medium was switched to DMEM with 1% FBS for 72 h; the medium was changed every 24 h. Then, cells were incubated in 2 ml of DMEM with 1% FBS containing various concentrations of AGEs (Biovision, USA) (25 *μ*g/ml, 50 *μ*g/ml, 100 *μ*g/ml, 200 *μ*g/ml, and 400 *μ*g/ml) and different time periods (6, 12, 24, and 48 hours) or adenovirus transfection. For some experiments, differentiated H9c2 cells were treated with Diphenyliodonium (DPI, antioxidants, 10 *μ*M), a selective ROCK inhibitor Y-27632 (1 *μ*M), and an actin depolymerizer agent Cytochalasin D (1 *μ*M) (Merck, Germany), all drugs being added 1 h before AGEs administration and throughout the AGEs treatment period. For adenovirus transfection, cells were transduced with a PFN-1 shRNA adenovirus (Adv-PFN-1) labeled with green fluorescent protein (GFP) or an empty adenoviral construct labeled with GFP (Adv-Control) (Hanbio Company, China) as control at a multiplicity of infection (MOI) of 40 for 4 hours. After transfection, the supernatant was replaced with DMEM with 1% FBS for another 20 h and then incubated with AGEs for further study.

### 2.2. Intracellular ROS Detecting and Measurement of Cell Size and Apoptosis

Intracellular ROS level was detected as described in our previous study [28]. To visualize the cell size change, cells were observed using a light microscope (Nikon, Japan) after different treatments. Cell apoptosis detection was carried out using a Hoechst 33258 kit (Beyotime Company, China) according to the manufacturer's protocol. Briefly, after each treatment, the cells were washed twice with cooled PBS. Then, the cells were incubated with 1 ml Hoechst 33258 (5 *μ*g/ml) for 5 min in the dark. After incubation, the Hoechst 33258 was removed and the cells were washed twice with PBS. The apoptosis nucleus was visualized under a fluorescent microscope (Nikon, Japan).

### 2.3. Immunofluorescent Cell Staining

The actin cytoskeleton was analyzed by detection of the structure of F-actin using the Leica TCS SP8 & MP confocal scanning microscope (Leica, Germany) as we previously described [28].

### 2.4. Quantitative Real-Time Polymerase Chain Reaction (qRT-PCR)

Total mRNA from cell samples was extracted using TRIzol reagent (Takara, Japan) according to the manufacturer's protocol. cDNA was generated from total RNA using a RT reagent kit (Takara, Japan). Amplification was performed by using 7500 Real-Time PCR Systems (Applied Biosystems, USA) with SYBR real-time PCR kit (Takara, Japan); the amplification procedure was performed with an initial step at 95°C for 30 s and 40 cycles of denaturation at 95°C for 5 s and annealing at 60°C for 34 s for each target gene. Results were expressed as the ratios of target genes against glyceraldehyde phosphate dehydrogenase (GAPDH) mRNA. All primers were designed and synthesized by Shanghai Sangon Company (Shanghai, China). The primers used in amplification were as follows: PFN-1-F, 5′-GCCTACATCGACAGCCTTATG-3′, PFN-1-R, 5′-TCTTTGCCTACCAGGACACC-3′; atrial natriuretic factor (ANF)-F, 5′-CTCCGATAGATCTGCCCTCTTGAA-3′, ANF-R, 5′-GGTACCGGAAGCTGTTGCAGCCTA-3′; *β*-myosin heavy chains (*β*-MHC)-F, 5′-CCAGAAGCCTCGAAATGTC-3′, *β*-MHC-R, 5′-CTTTCTTTGCCTTGCCTTTGC-3′; GAPDH-F, 5′-CCATGTTCGTCATGGGTGTGAACCA-3′, GAPDH-R, 5′-GCCAGTAGAGGCAGGGATGATGTTC-3′.

### 2.5. Western Blot

Total protein from cell samples was extracted and measured using a bicinchoninic acid (BCA) Protein Assay Kit (Beyotime, Jiangsu, China). After being heated at 95°C for 5 min, lysates were separated by SDS-PAGE gels and then transferred to PVDF membranes. The membranes were blocked with 5% fat-free milk in TBST buffer for 90 min and subsequently incubated with corresponding antibodies as follows: RAGE, RhoA, ROCK1, and ROCK2 (1 : 200, 1 : 800, 1 : 2000, and 1 : 600, resp.; Santa Cruz), cleaved caspase-3 and GAPDH (1 : 1000 and 1 : 4000, resp.; Cell Signaling), and PFN-1 (1 : 7500; Abcam) at 4°C overnight. After incubation, the membranes were washed three times and then incubated with HRP-conjugated secondary goat anti-rabbit or mouse IgG for 1 hour. The signal was detected and ratios of the target protein against GAPDH control were calculated using the Bio-Rad ChemiDoc XRS+ Imaging System (Bio-Rad Biosciences).

### 2.6. Statistical Analysis

Data were expressed as mean ± SD. Statistical analysis was performed using GraphPad Prism (version 6.0) software by unpaired Student's *t*-test and one-way ANOVA. A value of *P* < 0.05 was considered statistically significant.

## 3. Results

### 3.1. The Changes of PFN-1 Expression in AGEs-Treated H9c2 Cells

**Figure 1:**
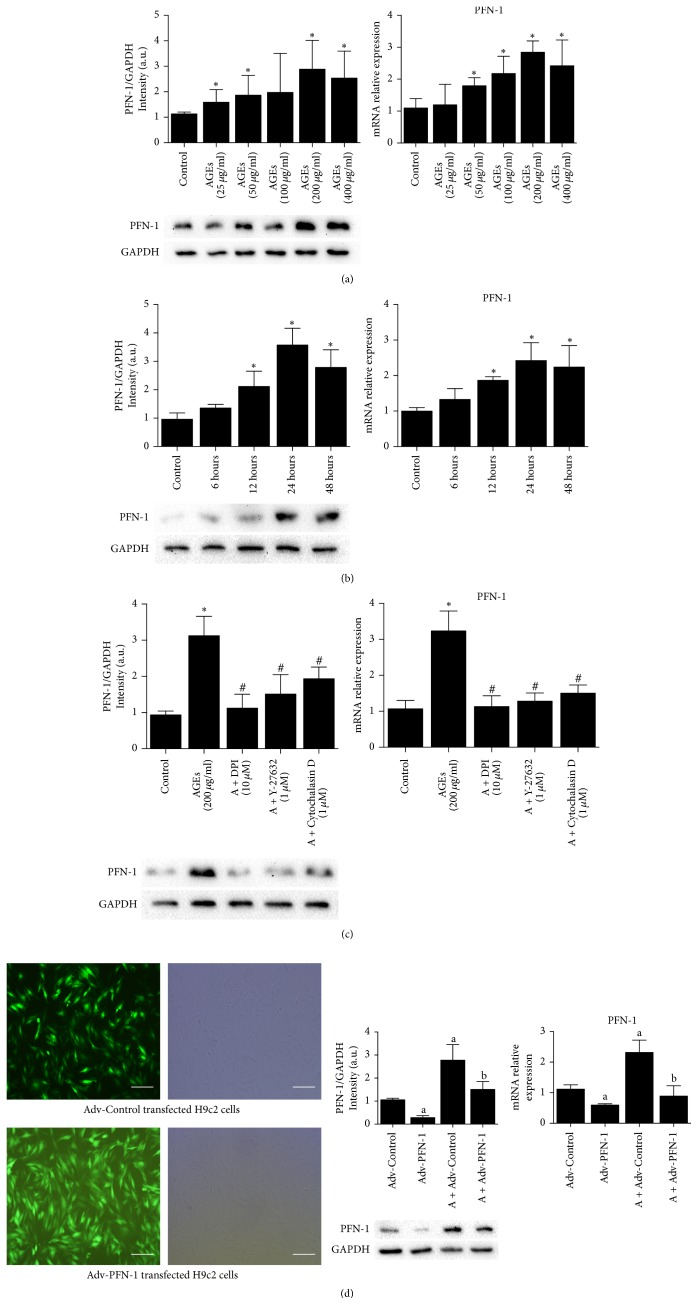
The changes of PFN-1 expression in H9c2 cells induced by AGEs. (a) Dose study: PFN-1 protein and mRNA expression in H9c2 cells incubated with different doses of AGEs for 24 hours. (b) Time study: PFN-1 protein and mRNA expression in H9c2 cells incubated with AGEs at a dose of 200 *μ*g/ml for different times. (c) The effect of different inhibitors on AGEs-induced (200 *μ*g/ml, 24 hours) PFN-1 protein and mRNA expression. (d) Left: representative images show PFN-1 Adv-shRNA adenovirus transfection probed by GFP (magnification: 20x; scale bar: 200 *μ*m). Middle and right: the effect of PFN-1 Adv-shRNA on PFN-1 protein and mRNA expression incubated with or without AGEs (200 *μ*g/ml, 24 hours). Data was expressed as mean ± SD, *n* = 3–6. ^*∗*^*P* < 0.05 versus control; ^#^*P* < 0.05 versus AGEs; ^a^*P* < 0.05 versus Adv-Control, ^b^*P* < 0.05 versus A + Adv-Control.

We first examined the expression of PFN-1 in differentiated H9c2 cells exposed to medium alone or AGEs. Compared with the control, treatment with AGEs at a dose of 50, 100, 200, or 400 *μ*g/ml significantly increased the mRNA and protein expression of PFN-1 for 24 hours (*P* < 0.05, Figure 1(a)). Furthermore, the time course study showed that AGEs at 200 *μ*g/ml had the most robust effect on the expression of PFN-1 at both mRNA and protein levels after 24 hours of treatment (Figure 1(b)). Thus, 200 *μ*g/ml AGE for 24 hours was selected for the further studies.

Further intervention experiments showed that the upregulated mRNA and protein expression of PFN-1 mediated by AGEs was blocked by treatment with antioxidants DPI (10 *μ*M), ROCK inhibitor Y-27632 (1 *μ*M), and actin depolymerizer Cytochalasin D (1 *μ*M) (*P* < 0.05, resp.) (Figure 1(c)). In addition, as shown in Figure 1(d), more than 90% of cells expressed green fluorescence, suggesting successful cell transfection with adenovirus, and in cells transfected with PFN-1 Ad-shRNA significantly reduced PFN-1 mRNA and protein expression under normal condition or AGEs treatment (*P* < 0.05, resp.).

### 3.2. Downregulation of PFN-1 Attenuates AGEs-Induced Cardiomyocytes Hypertrophy and Apoptosis

**Figure 2:**
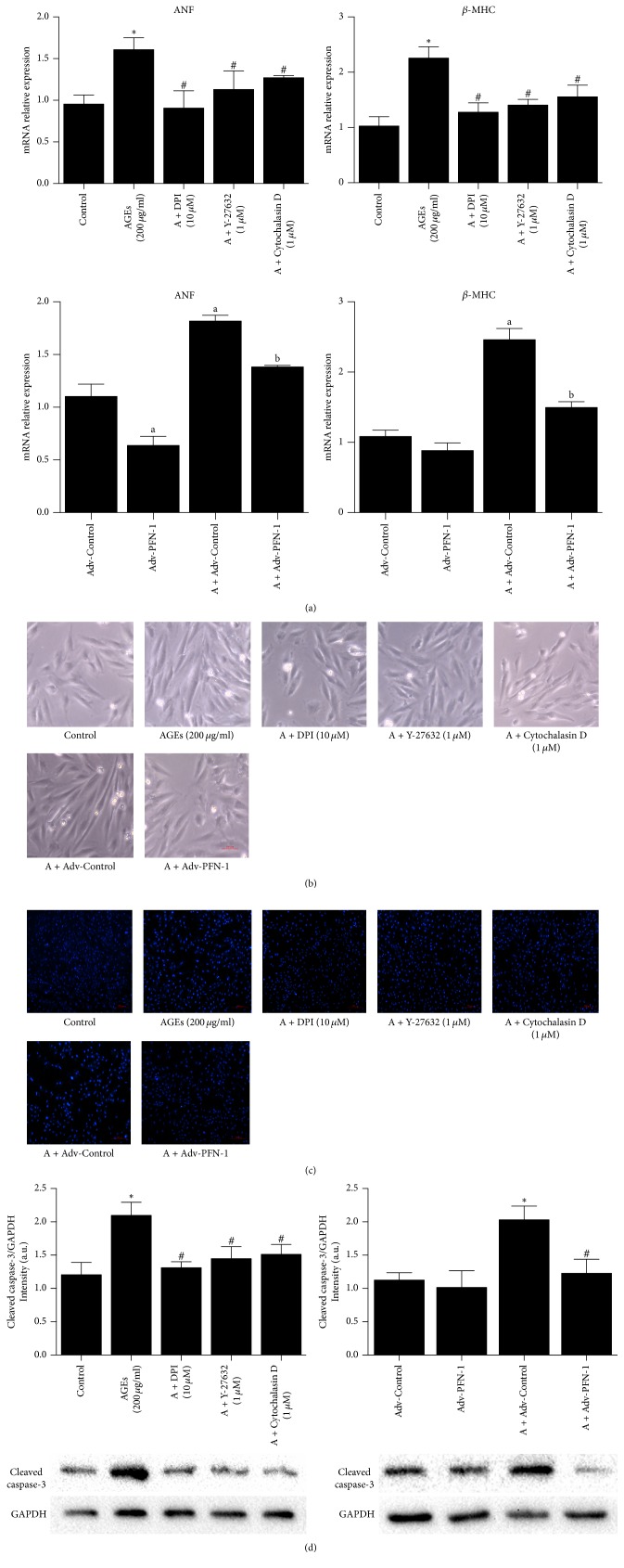
Effect of different inhibitors and PFN-1 Adv-shRNA adenovirus on AGEs-induced H9c2 cells hypertrophy and apoptosis. (a) Effect of different inhibitors and PFN-1 Adv-shRNA on AGEs-induced (200 *μ*g/ml, 24 hours) ANF and *β*-MHC mRNA expression. (b) Representative images show different inhibitors and PFN-1 Adv-shRNA adenovirus on cell size induced by AGEs (200 *μ*g/ml, 24 hours) (magnification: 40x; scale bar: 100 *μ*m). (c) Representative images show different inhibitors and PFN-1 Adv-shRNA adenovirus on apoptosis induced by AGEs (200 *μ*g/ml, 24 hours) (magnification: 20x; scale bar: 200 *μ*m). (d) Effect of different inhibitors and PFN-1 Adv-shRNA on cleaved caspase-3 protein expression induced by AGEs. Data were expressed as mean ± SD, *n* = 3. ^*∗*^*P* < 0.05 versus control; ^#^*P* < 0.05 versus AGEs; ^a^*P* < 0.05 versus Adv-Control; ^b^*P* < 0.05 versus A + Adv-Control.

It is well known that cardiomyocytes hypertrophy and apoptosis induced by AGEs plays an important role in the onset and development of DCM. In the present study, AGEs treatment markedly increased ANF and *β*-MHC mRNA expressions (two major hypertrophic genes) and cell size (Figures 2(a) and 2(b)) of H9c2 cells, suggesting pathological hypertrophy. Moreover, increased cardiomyocytes apoptosis was indicated by more bright spots in Hoechst 33258 staining and upregulated cleaved caspase-3 protein expression (Figures 2(c) and 2(d)). These effects were dramatically attenuated by PFN-1 silencing. Consistently, DPI, Y-27632, and Cytochalasin D also had a protective effect on AGEs-induced H9c2 cells hypertrophy and apoptosis (Figure 2), which occurred concomitantly with downregulated PFN-1 expression (Figure 1(c)).

### 3.3. Downregulation of PFN-1 Attenuates AGEs-Induced Redistribution and Formation of F-Actin in H9c2 Cells

**Figure 3:**
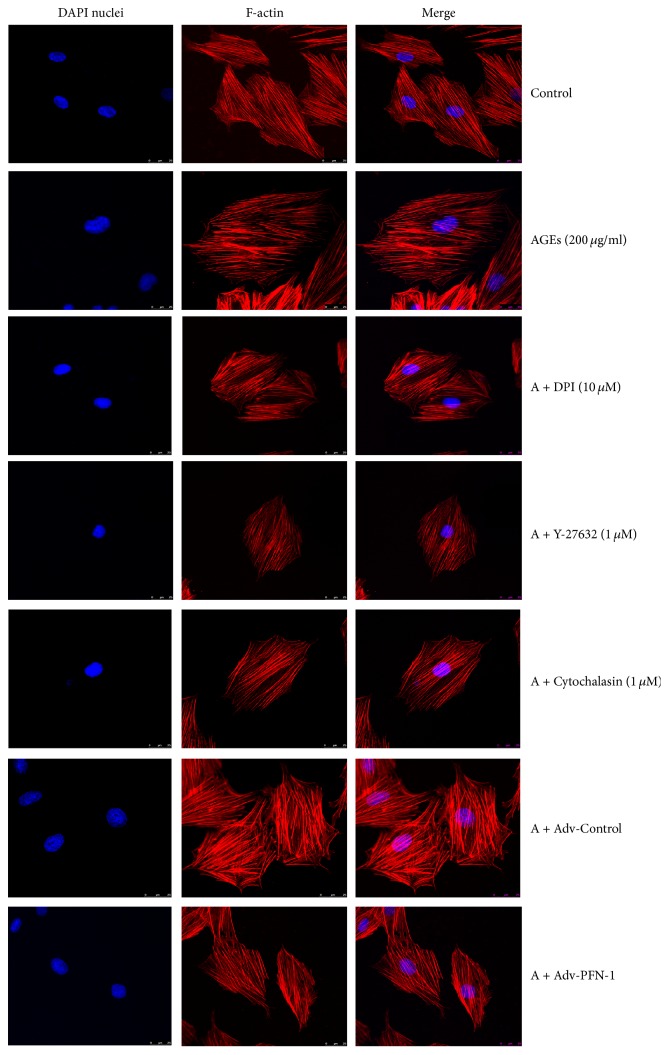
Effect of different inhibitors and Adv-PFN-1 shRNA adenovirus on AGEs-induced F-actin distribution in H9c2 cells. Representative images show different inhibitors and PFN-1 Adv-shRNA adenovirus on F-actin distribution induced by AGEs (200 *μ*g/ml, 24 hours). *n* = 3. DAPI nuclei (blue) and F-actin (red) (magnification: 60x; scale bar: 25 *μ*m).

The actin cytoskeleton forms the scaffold of cardiomyocytes, which is also required for regulating the signaling pathway and related gene expression under both physiological and pathological conditions [17–21]. Thus, the effect of AGEs on actin cytoskeletal organization in H9c2 cells was analyzed using fluorescence confocal microscopy to directly visualize actin filaments. In the control group, F-actin was well distributed and well organized in the cytoplasm, with 24 hours of AGEs (200 *μ*g/ml) treatment leading to a less well-structured F-actin formation (Figure 3). F-actin fiber morphology and distribution showed an observed change, as well as increased fluorescence intensity, suggesting elevated generation of F-actin. In contrast, downregulation of PFN-1 expression using adenovirus markedly improved F-actin fiber redistribution and decreased F-actin fluorescent intensity in response to AGEs. Likewise, in H9c2 cells treated with DPI, Y-27632 and Cytochalasin D also remarkably attenuated AGEs-induced F-actin fluorescent intensity and distribution in the cytoplasm.

### 3.4. The Alteration of ROS, RAGE, RhoA, and ROCK Expression in AGEs-Treated H9c2 Cells

**Figure 4:**
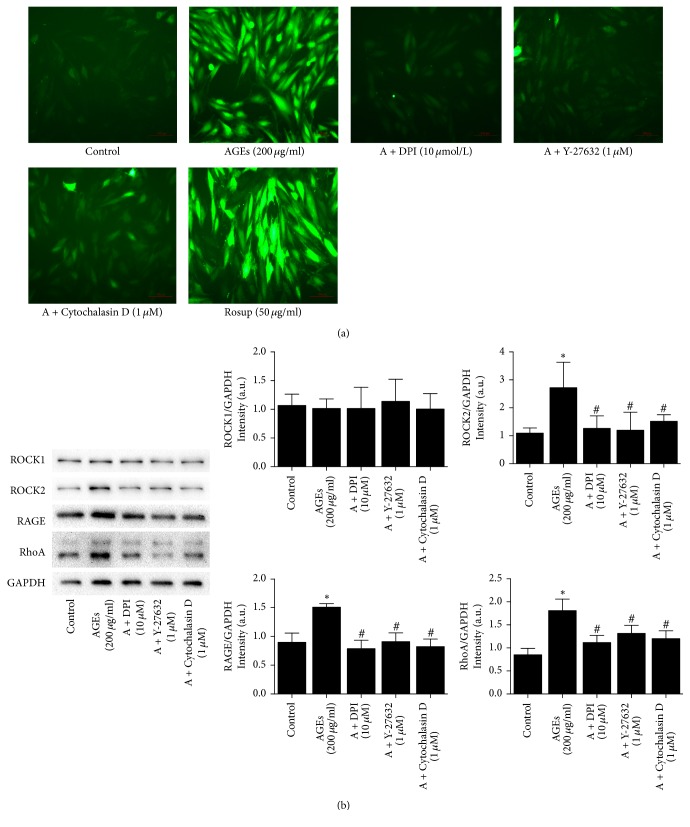
Effect of different inhibitors on intracellular ROS generation and RhoA/ROCK signaling pathway mediated by AGEs in H9c2 cells. (a) Representative images show different inhibitors on intracellular ROS generation induced by AGEs (200 *μ*g/ml, 24 hours). Rosup (50 *μ*g/ml) was used as a positive control (magnification: 20x; scale bar: 100 *μ*m). (b) Effect of different inhibitors on the protein expression of ROCK1, ROCK2, RAGE, and RhoA induced by AGEs. Data were expressed as mean ± SD, *n* = 3. ^*∗*^*P* < 0.05 versus control; ^#^*P* < 0.05 versus AGEs (200 *μ*g/ml, 24 hours).

Levels of ROS generation and the expression of RAGE, RhoA, and ROCK2 in H9c2 cells were increased after AGEs treatment compared to the control group (Figure 4). However, there was no difference in ROCK1 expression (Figure 4(b)). In contrast, in H9c2 cells, treatment with DPI, Y-27632, and Cytochalasin D markedly decreased ROS production as well as RAGE, RhoA, and ROCK2 expression (Figure 4).

## 4. Discussion

The main findings of the present study include the following: (1) AGEs significantly induced hypertrophy and apoptosis and upregulated PFN-1 expression in H9c2 cells, concomitantly with F-actin redistribution and increased formation of F-actin as well as RAGE, RhoA, and ROCK2 protein expression; (2) knockdown PFN-1 expression ameliorated AGEs-induced cardiomyocytes hypertrophy and apoptosis; (3) blockade of ROS production, ROCK activation, or treatment with an actin depolymerizer also remarkably improved AGEs-induced myocardial injury, which is in line with downregulated expression of PFN-1 and improved actin cytoskeleton alteration. Taken together, these data suggest for the first time that PFN-1 is involved in AGEs-induced cardiomyocytes hypertrophy and apoptosis, which may contribute to the onset and progression of DCM.

DCM is a distinct clinical entity, which may directly result from myocardial insult including cardiomyocytes hypertrophy and apoptosis under diabetic condition, different from other structural cardiac dysfunctions caused by atherosclerosis, hypertension, or congenital heart diseases [30, 31]. There are multiple risk factors contributing to the process of DCM, while AGEs have proven to be a pivotal factor in the development and progression of DCM [3]. Both intracellularly and extracellularly formed AGEs cause cardiac remodeling and result in heart failure through direct and indirect pathways. There is growing evidence that indirect pathways may play a predominant role in response to AGE by binding to its receptor RAGE, which increases the production of ROS, subsequently activating related ROS-dependent pathways including RhoA/ROCK signaling pathway [32–34]. Our present data shows that AGEs significantly promoted H9c2 cells hypertrophy and apoptosis* in vitro *in H9c2 cells, leading to remarkably elevated intracellular ROS levels and increased RAGE, RhoA, and ROCK2 expression. In support, treatment with DPI (antioxidants) and Y-27632 (ROCK inhibitor) significantly attenuated cardiomyocytes hypertrophy and apoptosis by inhibiting ROS accumulation and decreased RhoA and ROCK2 protein expression. These findings provide a complementary network demonstrating a key role of AGEs through increasing ROS generation and ROCK activation in the development of cardiomyocytes insults.

Previous studies indicated that PFN-1, an important actin-binding protein, could play an important role in pathological cardiac hypertrophy and activating hypertrophic signaling cascade. For instance, PFN-1 expression was increased in multiple hypertrophic phenotype models either* in vivo* or* in vitro*, such as ouabain, transverse aortic constriction (TAC), G*α*q-overexpressing and spontaneous hypertensive-induced ventricular hypertrophy in mice or rats [27, 35], and phenylephrine (PE) treated neonatal rat ventricular myocytes (NRVMs) [27]. Consistent with elevated PFN-1 expression in hypertrophic heart, downregulation of PFN-1 attenuated cardiac hypertrophy while PFN-1 overexpression or PFN-1 transgene promoted vascular and cardiac hypertrophy in SHRs [26]. More specifically, Kooij et al. [27] demonstrated that specific overexpression of PFN-1 using adenovirus in* Drosophila* heart and NRVMs resulted in a hypertrophic response partly through activation of ERK1/2 signaling cascade. These findings suggested that elevated PFN-1 was not only sufficient to induce a cardiac hypertrophic phenotype, but also necessary for hypertrophic response. However, detailed studies on the role of PFN-1 in AGEs-induced cardiomyocytes insult and whether silencing PFN-1 expression also has a protective effect in response to AGEs are still lacking. In our present study, we found that, in response to AGEs stimuli, PFN-1 expression was significantly elevated. Thus, we propose that PFN-1 may also contribute to AGEs-induced cardiomyocytes hypertrophy. To test this hypothesis, a specific adenovirus was used to silence PFN-1 expression. Transduction of PFN-1 Ad-shRNA in H9c2 cells with simultaneous AGEs treatment, the cardiac hypertrophy related gene expression including ANF and *β*-MHC, and cell size were significantly repressed. Interestingly, in addition to hypertrophic insult, we also found that downregulation of PFN-1 expression attenuated AGEs-induced H9c2 cells apoptosis, indicated by a decreased cleaved caspase-3 expression and Hoechst 33258 staining. These data suggest that elevated PFN-1 expression plays a crucial role in AGEs-induced cardiomyocytes hypertrophy and apoptosis and silencing PFN-1 expression has a protective effect.

First dynamic changes of actin cytoskeleton in cardiomyocytes are required for cardiac hypertrophy and failure [36, 37]. Previous studies demonstrated that actin polymerization, which is manifested by an increased ratio of F-actin to G-actin, and RhoA/ROCK activation were significantly increased in diabetic cardiomyocytes [22, 23]. RhoA/ROCK pathway is a well-known regulator of the actin cytoskeleton dynamics and plays an important role in pathological cardiac remodeling [38]. However, the link between RhoA/ROCK pathway and actin cytoskeletal alteration remains obscure in DCM. The results of the present study showed that an observed intracellular actin distribution, which was manifested by F-actin redistribution, as well as increased F-actin formation, was found in AGEs-treated H9c2 cells. In line, PFN-1 expression was increased whereas Y-27632-treated H9c2 cells, which is a common inhibitor for ROCK, significantly attenuated AGEs-induced cardiomyocytes hypertrophy and apoptosis. This was further accompanied by decreased PFN-1 expression and reorganized cytoskeleton alteration and decreased F-actin formation. In support, silencing PFN-1 expression using adenovirus significantly attenuated the AGEs-induced actin cytoskeletal reorganization and H9c2 cells hypertrophy as well as apoptosis. Further evidence for an association between AGEs and actin cytoskeleton alteration was also demonstrated in the present study using Cytochalasin D, an actin depolymerizer drug, mimicking the protective effect as Y-27632 and silencing PFN-1 expression on myocardial damage induced by AGEs. Taken together, these findings suggested that PFN-1 is not simply a necessary hypertrophic molecule but serves as a potent link between RhoA/ROCK pathway and actin cytoskeletal changes induced by AGEs.

## 5. Conclusion

In summary, PFN-1 may serve as a novel regulator that mediates the effects of AGEs-activated RhoA/ROCK signaling pathway on the redistribution of actin cytoskeleton, and inhibition of PFN-1 expression can protect against cardiomyocytes injury and cardiac dysfunction in diabetes. These findings suggested that profilin-1 might be a novel therapeutic target for prevention and treatment of cardiac injury in DCM.

## References

[1] Miki T., Yuda S., Kouzu H., Miura T. (2013). Diabetic cardiomyopathy: pathophysiology and clinical features. *Heart Failure Reviews*.

[2] Bando Y. K., Murohara T. (2014). Diabetes-related heart failure: does diabetic cardiomyopathy exist?. *Circulation Journal*.

[3] Bodiga V. L., Eda S. R., Bodiga S. (2014). Advanced glycation end products: role in pathology of diabetic cardiomyopathy. *Heart Failure Reviews*.

[4] Singh V. P., Bali A., Singh N., Jaggi A. S. (2014). Advanced glycation end products and diabetic complications. *Korean Journal of Physiology & Pharmacology*.

[5] Cooper M. E. (2004). Importance of advanced glycation end products in diabetes-associated cardiovascular and renal disease. *American Journal of Hypertension*.

[6] Nozynski J., Zakliczynski M., Konecka-Mrowka D. (2012). Advanced glycation end product accumulation in the cardiomyocytes of heart failure patients with and without diabetes. *Annals of Transplantation*.

[7] Van Heerebeek L., Hamdani N., Handoko M. L. (2008). Diastolic stiffness of the failing diabetic heart: importance of fibrosis, advanced glycation end products, and myocyte resting tension. *Circulation*.

[8] Kellow N. J., Coughlan M. T. (2015). Effect of diet-derived advanced glycation end products on inflammation. *Nutrition Reviews*.

[9] Uribarri J., Cai W., Sandu O., Peppa M., Goldberg T., Vlassara H. (2005). Diet-derived advanced glycation end products are major contributors to the body's AGE pool and induce inflammation in healthy subjects. *Annals of the New York Academy of Sciences*.

[10] Ko S.-Y., Lin I.-H., Shieh T.-M. (2013). Cell hypertrophy and MEK/ERK phosphorylation are regulated by glyceraldehyde-derived AGEs in cardiomyocyte H9c2 cells. *Cell Biochemistry and Biophysics*.

[11] Li S.-Y., Sigmon V. K., Babcock S. A., Ren J. (2007). Advanced glycation endproduct induces ROS accumulation, apoptosis, MAP kinase activation and nuclear O-GlcNAcylation in human cardiac myocytes. *Life Sciences*.

[12] Guo R., Liu W., Liu B., Zhang B., Li W., Xu Y. (2015). SIRT1 suppresses cardiomyocyte apoptosis in diabetic cardiomyopathy: an insight into endoplasmic reticulum stress response mechanism. *International Journal of Cardiology*.

[13] Brouwers O., de vos-Houben J. M. J., Niessen P. M. G. (2013). Mild oxidative damage in the diabetic rat heart is attenuated by glyoxalase-1 overexpression. *International Journal of Molecular Sciences*.

[14] Yamagishi S.-I., Fukami K., Matsui T. (2015). Evaluation of tissue accumulation levels of advanced glycation end products by skin autofluorescence: a novel marker of vascular complications in high-risk patients for cardiovascular disease. *International Journal of Cardiology*.

[15] Sveen K. A., Nerdrum T., Hanssen K. F. (2014). Impaired left ventricular function and myocardial blood flow reserve in patients with long-term type 1 diabetes and no significant coronary artery disease: associations with protein glycation. *Diabetes and Vascular Disease Research*.

[16] Nenna A., Spadaccio C., Lusini M., Ulianich L., Chello M., Nappi F. (2015). Basic and clinical research against advanced glycation end products (AGEs): New compounds to tackle cardiovascular disease and diabetic complications. *Recent Advances in Cardiovascular Drug Discovery*.

[17] Squire J. M. (1997). Architecture and function in the muscle sarcomere. *Current Opinion in Structural Biology*.

[18] Sequeira V., Nijenkamp L. L. A. M., Regan J. A., Van Der Velden J. (2014). The physiological role of cardiac cytoskeleton and its alterations in heart failure. *Biochimica et Biophysica Acta (BBA) - Biomembranes*.

[19] Cooper G. (2006). Cytoskeletal networks and the regulation of cardiac contractility: microtubules, hypertrophy, and cardiac dysfunction. *American Journal of Physiology-Heart and Circulatory Physiology*.

[20] Kostin S., Hein S., Arnon E., Scholz D., Schaper J. (2000). The cytoskeleton and related proteins in the human failing heart. *Heart Failure Reviews*.

[21] Frank D., Kuhn C., Katus H. A., Frey N. (2006). The sarcomeric Z-disc: a nodal point in signalling and disease. *Journal of Molecular Medicine*.

[22] Soliman H., Gador A., Lu Y.-H., Lin G., Bankar G., MacLeod K. M. (2012). Diabetes-induced increased oxidative stress in cardiomyocytes is sustained by a positive feedback loop involving Rho kinase and PKC*β*2. *American Journal of Physiology-Heart and Circulatory Physiology*.

[23] Lin G., Craig G. P., Zhang L. (2007). Acute inhibition of Rho-kinase improves cardiac contractile function in streptozotocin-diabetic rats. *Cardiovascular Research*.

[24] Lai D., Gao J., Bi X. (2017). The Rho kinase inhibitor, fasudil, ameliorates diabetes-induced cardiac dysfunction by improving calcium clearance and actin remodeling. *Journal of Molecular Medicine*.

[25] Witke W. (2004). The role of profilin complexes in cell motility and other cellular processes. *Trends in Cell Biology*.

[26] Zhao S.-H., Qiu J., Wang Y. (2013). Profilin-1 promotes the development of hypertension-induced cardiac hypertrophy. *Journal of Hypertension*.

[27] Kooij V., Viswanathan M. C., Lee D. I. (2016). Profilin modulates sarcomeric organization and mediates cardiomyocyte hypertrophy. *Cardiovascular Research*.

[28] Li Z., Zhong Q., Yang T., Xie X., Chen M. (2013). The role of profilin-1 in endothelial cell injury induced by advanced glycation end products (AGEs). *Cardiovascular Diabetology*.

[29] Yang D., Liu W., Ma L. (2017). Profilin-1 contributes to cardiac injury induced by advanced glycation end-products in rats. *Molecular Medicine Reports*.

[30] Khavandi K., Khavandi A., Asghar O. (2009). Diabetic cardiomyopathy - a distinct disease?. *Best Practice & Research Clinical Endocrinology & Metabolism*.

[31] Bugger H., Abel E. D. (2014). Molecular mechanisms of diabetic cardiomyopathy. *Diabetologia*.

[32] Shimokawa H., Sunamura S., Satoh K. (2016). RhoA/Rho-Kinase in the cardiovascular system. *Circulation Research*.

[33] Amano M., Nakayama M., Kaibuchi K. (2010). Rho-kinase/ROCK: a key regulator of the cytoskeleton and cell polarity. *Cytoskeleton*.

[34] Shimizu T., Liao J. K. (2016). Rho kinases and cardiac remodeling. *Circulation Journal*.

[35] Zhao S.-H., Gao H.-Q., Ji X. (2013). Effect of ouabain on myocardial ultrastructure and cytoskeleton during the development of ventricular hypertrophy. *Heart and Vessels*.

[36] Hein S., Kostin S., Heling A., Maeno Y., Schaper J. (2000). The role of the cytoskeleton in heart failure. *Cardiovascular Research*.

[37] Zeidan A., Javadov S., Karmazyn M. (2006). Essential role of Rho/ROCK-dependent processes and actin dynamics in mediating leptin-induced hypertrophy in rat neonatal ventricular myocytes. *Cardiovascular Research*.

[38] Nunes K. P., Rigsby C. S., Webb R. C. (2010). RhoA/Rho-kinase and vascular diseases: what is the link?. *Cellular and Molecular Life Sciences*.

